# Reconstruction of gene co-expression network from microarray data using local expression patterns

**DOI:** 10.1186/1471-2105-15-S7-S10

**Published:** 2014-05-28

**Authors:** Swarup Roy, Dhruba K Bhattacharyya, Jugal K Kalita

**Affiliations:** 1 Dept of Information Technology, North Eastern Hill University, Umshing, Shillong 793 022, Meghalaya, India; 2 Dept of Computer Science & Engineering, Tezpur University, Napaam 784 028, Assam, India; 3 Dept of Computer Science, University of Colorado, Colorado Springs, USA

## Abstract

**Background:**

Biological networks connect genes, gene products to one another. A network of co-regulated genes may form gene clusters that can encode proteins and take part in common biological processes. A gene co-expression network describes inter-relationships among genes. Existing techniques generally depend on proximity measures based on global similarity to draw the relationship between genes. It has been observed that expression profiles are sharing local similarity rather than global similarity. We propose an expression pattern based method called **GeCON **to extract **Ge**ne **CO**-expression **N**etwork from microarray data. Pair-wise supports are computed for each pair of genes based on changing tendencies and regulation patterns of the gene expression. Gene pairs showing negative or positive co-regulation under a given number of conditions are used to construct such gene co-expression network. We construct co-expression network with signed edges to reflect up- and down-regulation between pairs of genes. Most existing techniques do not emphasize computational efficiency. We exploit a fast correlogram matrix based technique for capturing the support of each gene pair to construct the network.

**Results:**

We apply GeCON to both real and synthetic gene expression data. We compare our results using the DREAM (*Dialogue for Reverse Engineering Assessments and Methods*) Challenge data with three well known algorithms, viz., ARACNE, CLR and MRNET. Our method outperforms other algorithms based on *in silico *regulatory network reconstruction. Experimental results show that GeCON can extract functionally enriched network modules from real expression data.

**Conclusions:**

In view of the results over several *in-silico *and real expression datasets, the proposed GeCON shows satisfactory performance in predicting co-expression network in a computationally inexpensive way. We further establish that a simple expression pattern matching is helpful in finding biologically relevant gene network. In future, we aim to introduce an enhanced GeCON to identify Protein-Protein interaction network complexes by incorporating variable density concept.

## Background

Cellular processes constitute complex systems and cannot be described using a simplistic view. To fully understand the functioning of cellular processes, it is not enough to simply assign functions to individual genes, proteins and other cellular macro-molecules. Biological networks depicting interactions among components present an integrated look at the dynamic behaviour of the cellular system. Biological networks may be categorized [1] as *metabolic pathways, signal transduction pathways, gene regulatory networks *and *protein-protein interaction (PPI) networks *[2]. The advent of micro-array technology enabled the system biologist to study the dynamic behaviour of genes in multiple conditions. Due to the availability of large collections of gene expression data, it is now possible to reconstruct or reverse-engineer the cellular system *in-silico*.

A gene co-expression network (CEN) is a collection of genes in a cell which interact with each other and with other molecules in the cell such as proteins or metabolites, thereby governing the rates at which genes in the network are transcribed into mRNA. A CEN is normally represented as an undirected graph, where a node represents a gene or gene product and an undirected edge represents a significant co-expression relationship [3,4] between the genes considering a series of gene expression measurements. On the other hand, a Gene Regulatory Network (GRN) is a directed graph, where a node represents a gene and a directed edge represents a biochemical process such as a reaction, transformation, interaction, activation or inhibition. Compared to a GRN, a CEN does not attempt to draw direct causal relationships among the participating genes in the form of directed edges. A module extracted from a co-expression network [5] may contain co-regulated gene clusters which interact among themselves and take part in a common biological process.

A number of techniques have been proposed for genetic network construction [6-12]. Many approaches use statistical, machine learning or soft-computing techniques [7] as discovery tools.

Network models such as Bayesian [13] and boolean networks [14] are used to infer interrelationships among genes. Kwon et al. [15] extract gene regulatory relationships for cell cycle-regulated genes with activation or inhibition between gene pairs. Regulatory relationships have also been deduced from correlation of co-expressions, between DNA-binding transcription regulators and target genes, by using a probabilistic expression model [16].

Mitra et al. [8] propose a bi-clustering technique to extract simple gene interaction networks. They use continuous column multi-objective evolutionary bi-clustering to extract rank correlated gene pairs. Such pairs are used to construct the gene network for generating relationship between a transcription factor and its target's expression level. Jung and Cho [9] propose an evolutionary approach for construction of gene (interaction) networks from gene expression time-series data. It assumes an artificial gene network and compares it with the reconstructed network from the gene expression time-series data generated by the artificial network. Next, it employs real gene expression time-series data to construct a gene network by applying the proposed approach.

Mutual information [17,18] or correlation [6,10-12] based approaches have been proposed for extracting genetic networks. It has been observed that two genes with high mutual information are non-randomly associated with each other with biological significance. Butte et al. [18] compute comprehensive pair-wise mutual information for all genes in an expression dataset. By picking a threshold for mutual information (MI) and using only associations at or above the threshold, they construct what are called Relevance Networks (RN). Followed by RN a number of promising techniques have been proposed so far. Some of the well known algorithms are CLR [19], ARACNE [20] and MRNET [21]. The CLR algorithm modifies the MI score based on the empirical distribution of all MI scores. The ARACNE algorithm filters out indirect interactions from triplets of genes with the data processing inequality. MRNET uses an iterative feature selection method based on a maximum relevance/minimum redundancy criterion.

From biological point of view, expression patterns convey significant meaning. Two genes happen to be biologically associated, if their expression profiles show pattern similarity. As a result, existing gene expression analysis techniques give importance directly or indirectly to the pattern based similarity. Below we present a brief discussion on various expression patterns generally observed in the gene expression data.

### Patterns in expression profiles

**Figure 1:**
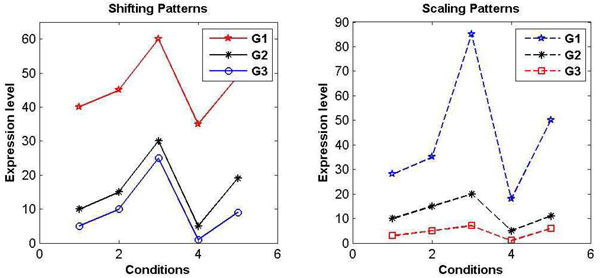
**Shifting and scaling patterns**. In shifting patterns, expression values maintains a additive distance whereas scaling patterns show multiplicative distance between the expression values of two expression profiles. Such patterns are also termed as positive or co-expressed patterns.

Profile plots of gene expression data revels a number of interesting patterns in the expression. From biological point of view, patterns play an important role in discovering functions of genes, disease targets or gene interactions. Scaling and Shifting [22] are the patterns that commonly discussed in majority of the literatures. In shifting patterns [23] the gene profiles show similar trends, but distance-wise, they may be away from each other (see Figure 1).

In terms of expression values, gene patterns follow an additive distance between them. Formally, shifting pattern can be defined as follows.

Given two gene expression profile *G_i _*= {*E*_*i*1_, *E*_*i*2_, · · ·, *E*_ik_} and *G_j _*= {*E*_*j*1_, *E*_*j*2_, *· · · , E*_jM_} with *M *expression values, a profile is called as shifted pattern, if expression value *E_ik _*can be related with *E_jk _*with constant additive factor *α_k _*under *k^th ^*condition. This can be written as follows.

(1) $$E_{ik}=E_{jk}+\alpha_{k},\;\text{for}\;k=1\text{ to }M$$

Similarly, scaling patterns in gene expression follow roughly a multiplicative distance between the patterns. A profile is called as scaling pattern, if expression value *E_ik _*can be related with *E_jk _*with constant multiplicative factor *β_k _*under *k^th ^*condition. Scaling pattern can be defined as:

(2) $$E_{ik}=E_{jk}\times \beta_{k},\text{ for }k=1\text{ to }M$$

As shown in Figure 1, values of *G*_2 _are roughly three times larger than those of *G*_3_, and values of *G*_1 _are roughly three times larger than those of *G*_2_. In nature, it may happen that due to different environmental stimuli or conditions, the pattern *G*_3 _responds to these conditions similarly, although *G*_1 _is more responsive or more sensitive to the stimuli than the other two.

Most often the patterns in Figure 1 are termed as co-expressed genes having similar expression patterns. Co-expressed patterns signify positive regulation relationship between the genes. In such patterns increase or decrease in expression level of gene *G_i _*leads to increase or decrease in expression level of gene *G_j _*respectively under the same conditions or time points.

**Figure 2:**
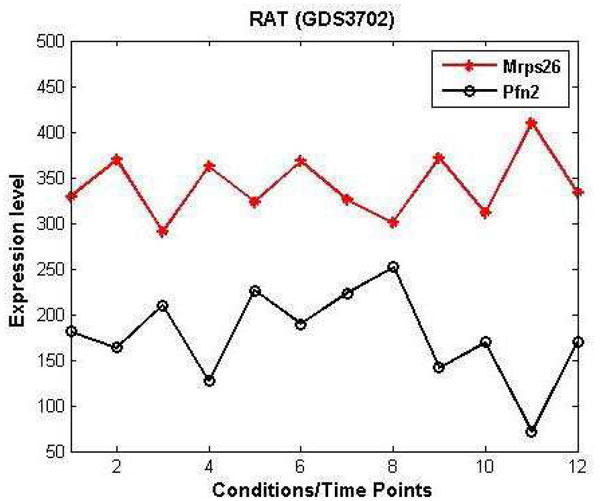
**Negative or inverted regulation patterns**. Expression profiles taken from *Mrps26 *and *Pfn2 *RAT genes clearly showing negative regulation i.e., increase in expression level of one gene leads to decrease in the expression level of other and vice versa.

**Figure 3:**
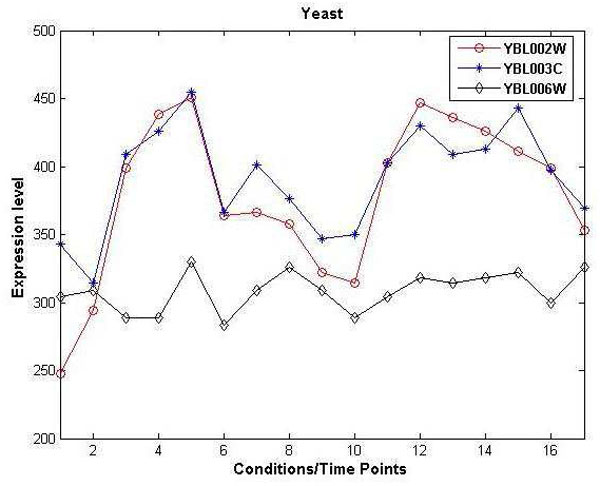
**Mix regulation patterns**. Yeast genes *YBL002W, YBL006W *and *YBL003C *showing both positive and negative expression patterns with respect to certain time-points or conditions. *YBL002W *and *YBL003C *are co-regulated and *YBL006W *shows inverted behaviour with respect to other two genes.

We further note that two genes may be related to each other even when their expression patterns show negative or inverted behaviour [24]. In Figure 2, expression patterns of Rat genes *Mrps26 *and *Pfn2*, taken from the NCBI dataset, GDS3702, clearly show negative behaviour. Gene ontology suggests that both are responsible for regulation of interferon-beta production. Again, we easily observe that in the Yeast datsets given in [25], genes *YBL002W *and *YBL003C *have a similar pattern and gene *YBL006W *has an inverted behaviour with respect to the other two genes. If we observe Figure 3 more closely, we see that expression patterns also share mixed regulation (i.e., both positive and negative). As suggested by Gene Ontology all three genes are involved in nucleosome organization, protein-DNA complex sub-unit organization, chromatin and chromosome organization and cellular macro-molecular subunit organization. A group of genes may share a combination of both positive and negative co-regulation under a few conditions or at a few time points. A majority of existing approaches capture genes with similar tendencies as co-expression but ignores patterns like the ones we discuss above. In computing similarity, many well-known techniques do not consider positive- or negative-regulation patterns as presenting co-expression or co-regulation with associated biological significance.

While computing association between a pair genes in a network, most existing techniques extract network using global similarity measures such as correlation or mutual information. Correlation measures ineffective in handling scaling and shifting patterns, where shape of two expression patterns are similar although values are not equal. Such patterns may affect the correlation measure in drawing out true associations among genes. Mutual information (MI) based techniques are effective alternatives to correlation measures. MI works well with co-expressed or positively regulated patterns. However, it fails in handling gene profile with negative and mixed patterns. Moreover, MI discretizes the expression values before computation that may lead to information loss. Thus, pairwise correlation or mutual information may not able to reveal proper relationships. Further, it has been observed that two expression profiles may match each other under some conditions or samples. Existing approaches generally compute similarity considering expression values in all dimensions. As a result, correlation score sometimes penalized due to mismatch in the expression values of two genes under some conditions. To handle such situations bi-clustering techniques [26] are found suitable in drawing relationship between a pair [8] of genes in a network. Bi-clustering attempts to find subset of genes under subset of conditions. On the other hand in a network, associations are explored between a pair of genes not within a group of genes. As a result, bi-clustering may not be an effective way to be applied while constructing co-expression network. Moreover, bi-clustering based techniques are normally computationally expensive in nature.

In our work, we demonstrate that a simple pattern matching based technique can give promising outcomes. We capture pair-wise similarity purely by pattern matching that can handle all types of patterns as discussed above. We consider both positive- and negative-regulation as co-regulation. Unlike available measures, we use a support based approach to compute similarity between two expression patterns and include the case where two genes are similar only under some conditions. Available techniques for finding co-expression networks mostly discover only limited associations among the genes without any regulation information. Since creating a co-expression network is a preliminary step towards gene regulatory network discovery, we use signed edges between the genes to represent positive- and negative-regulations, an important component in gene regulatory networks. Majority of the techniques ignore an important aspect i.e. computational costs. Computing correlation or mutual information for all possible pairs is a costly affair. Over the decade, only a few approaches have been developed to discover gene co-expression networks most of which are expensive in nature. We give due emphasis on development of a computationally effective network reconstruction technique. We compute the similarity between pair of genes using a fast one-pass support count based approach. Strong support between a pair of genes represent strong association between them. Gene pairs showing high support, i.e., high pattern similarity are used to construct a gene co-expression network. We apply our approach to several synthetic and real expression datasets. We assess our results from real datasets by evaluating the network modules extracted from the network against biologically significant Gene Ontology (GO) terms associated with a group.

## Results and discussion

This section provides details of experiments conducted, the datasets used and validation of the results. We apply GeCON to real and synthetic gene expression data consisting of publicly available seven benchmark gene expression datasets and thirteen *in silico *datasets from the DREAM (*Dialogue for Reverse Engineering Assessments and Methods*) Challenges.

### Input parameters

During our experiments, we observe that higher number of edge (discussed below) matches between a pair of gene expressions give more significant outcomes. Thus, in most experiments, we try to keep the value of *θ *above 50% of the total number of edges present in the dataset. In order to calculate similarity between two expression profiles in terms of degree of fluctuation, we achieve good results with *τ *ranging between 15 to 25.

### *In silico* dataset

We use the DREAM Challenge data, available in [27], for *in silico *regulatory network construction. Dream3 and Dream4 are the two Challenges for which data are available. Dream3 involves fifteen benchmark datasets, five each of various sizes (10, 50 and 100). The structures of the benchmark networks are obtained by extracting modules from real biological networks. At each size, two of the networks are extracted from the regulatory networks of *E. coli *and Yeast. Dream4 dataset is very similar to the Dream3 dataset, containing a total of 10 networks, five each of size 10 and 100. The *in silico *datasets generated based on [27] for our experiments are characterized in Table 1.

**Table 1:** *In silico *DREAM challenge datasets

Challenges	Dataset	*In silico *network	Size of the network
	D1	Ecoli1	10
	D2	Ecoli2	10
	D3	Ecoli1	50
	D4	Ecoli2	50
Dream3	D5	Yeast1	10
	D6	Yeast2	10
	D7	Yeast1	50
	D8	Yeast2	50

	D9	insilico1	10
	D10	insilico2	10
Dream4	D11	insilico3	10
	D12	insilico1	100
	D13	insilico2	100

Brief description about DREAM challenge synthetic datasets generated using Marbach platform. Network size indicates the number of genes participated in the network.

We compare our predictions with three well-known gene regulatory network reconstruction algorithms, ARACNE [20], CLR [19] and MRNET [21]. R implementation of the three algorithms is available in [28]. For the three algorithms, we use the parameters as used in [28]. Prediction effectiveness is compared against the *in slico *networks given in Marbach platform [27] using three different metrics for evaluating accuracy: AUPvR (Area under Precision vs Recall curve), AUROC (Area under Receiver Operating Characteristics curve) and *F_β_* score. ROC curves are commonly used to evaluate prediction results. However, ROC curves may not be the appropriate measure when a dataset contains large skews in the class distribution, which is commonly the case in transcriptional network inference. As an alternative, precision vs. recall (PvR) curves are used for measuring prediction accuracy [29]. The PvR curve may be more sensitive when there is a much larger negative set than the positive set. Computing the area under the curve (AUC) of a ROC or PvR curve is a way to reduce ROC or PvR performance to a single value representing expected performance. A compact representation of the PvR diagram is the maximum and/or the average F score [30], which is the harmonic average of precision and recall. The general formula for F score with respect to a non-negative *β *value is:

(3) $$F_{\beta }=\left(1+\beta^{2}\right)\frac{precision.recall}{\left(\beta^{2}.precision\right)+recall}.$$

**Figure 4:**
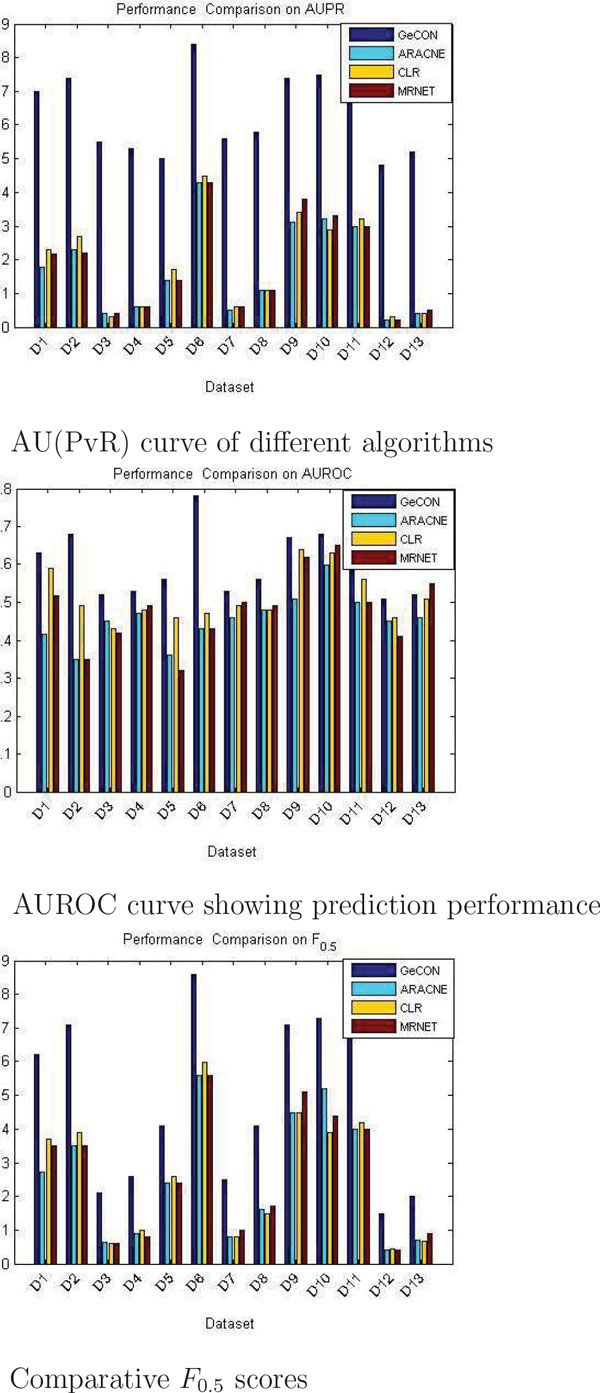
**Performance comparison of four algorithms on in silico dataset**. Results show prediction accuracy of GeCON compare to other three algorithms based on 13 *in-silico *DREAM challenge data. Performances are measured using precision-recall curve, ROC curve and F-score. In all cases GeCON exhibits superior performance.

Two commonly used *F *measures are the *F*_2 _measure, which weights recall higher than precision, and the *F*_0.5 _measure, which puts more emphasis on precision than recall. The F-score estimates the effectiveness of retrieval assuming recall is *β *times more important than precision. In our experiments we preferred *F*_0.5 _score. The effectiveness of prediction by GeCON on all the datasets compared to other algorithms are shown in Figure 4. An average percentage improvement of GeCON over other algorithms along with performance scores are also presented in Table 2. In terms of AUPR, GeCON achieves more than 200 times better performance than other algorithms. Similarly for other scores we can easily observe performance improvement of GeCON compare to other algorithms.

**Table 2:** Performance scores of different algorithms

Dataset	AUPR				AUROC				F Score			
	
	GeCON	ARACNE	CLR	MRNET	GeCON	ARACNE	CLR	MRNET	GeCON	ARACNE	CLR	MRNET
D1	0.7	0.177	0.23	0.218	0.63	0.416	0.59	0.517	0.62	0.271	0.37	0.35

D2	0.74	0.229	0.27	0.22	0.68	0.35	0.49	0.35	0.71	0.35	0.39	0.35

D3	0.55	0.04	0.03	0.04	0.52	0.45	0.43	0.42	0.21	0.063	0.06	0.06

D4	0.53	0.06	0.06	0.06	0.53	0.47	0.48	0.49	0.26	0.09	0.1	0.08

D5	0.5	0.14	0.17	0.14	0.56	0.36	0.46	0.32	0.41	0.24	0.26	0.24

D6	0.84	0.43	0.45	0.43	0.78	0.43	0.47	0.43	0.86	0.56	0.6	0.56

D7	0.56	0.05	0.06	0.06	0.53	0.46	0.49	0.5	0.25	0.08	0.08	0.1

D8	0.58	0.11	0.11	0.11	0.56	0.48	0.48	0.49	0.41	0.16	0.15	0.17

D9	0.74	0.31	0.34	0.38	0.67	0.51	0.64	0.62	0.71	0.45	0.45	0.51

D10	0.75	0.32	0.29	0.33	0.68	0.6	0.63	0.65	0.73	0.52	0.39	0.44

D11	0.74	0.3	0.32	0.3	0.67	0.5	0.56	0.5	0.71	0.4	0.42	0.4

D12	0.48	0.02	0.03	0.02	0.51	0.45	0.46	0.41	0.15	0.042	0.044	0.041

D13	0.52	0.04	0.04	0.05	0.52	0.46	0.51	0.55	0.2	0.069	0.066	0.09

Average	0.633	0.171	0.184	0.181	0.603	0.456	0.514	0.480	0.479	0.253	0.26	0.260

Performance improvement of GeCON (%)over		269.72	242.91	249.024		32.07	17.18	25.50		89.07	84.31	83.72

The performance scores of four algorithms and the performance improvement of GeCON compare to other algorithms in terms of three measures namely, AUPR, AUROC and F-score.

From the figures it is evident that GeCON outperforms all other algorithms in terms of network prediction on all three scores. In case of dataset *D*6, GeCON achieves a very high AU(PvR) score of .84 and AUROC of .78 and *F_β _*score of .86. Other algorithms exhibit consistent and almost similar trends in all experiments. To justify our claim of one-pass nature of GeCON, which is fast in general, we perform execution time comparison of GeCON with ARACNE. Due to unavailability of executable codes of all other target algorithms on a Java platform, we used only the Java version of the original ARACNE code (http://wiki.c2b2.columbia.edu/califanolab/index.php/Software/ARACNE) for comparison with GeCON.

**Figure 5:**
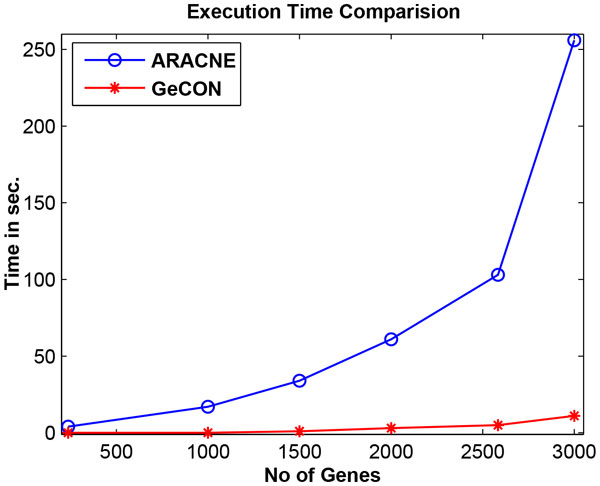
**Execution time comparison**. Due to GeCON's one-pass nature it consumes less time compare to ARCNE. Several synthetic expression datasets are generated using Marbach platform with varying number of gene expressions and tested in terms of CPU time requirements.

We generate different *in-silico *expression datasets using the Marbach platform [27] by varying the number of genes, keeping the number of time points at 50. The results given in Figure 5 clearly show that GeCON is much faster than ARACNE.

### Real datasets

We analyze the results from various real datasets for biological significance in terms of the GO annotation database. The details of the datasets are presented in Table 3.

**Table 3:** Short description of the datasets

Organism	Dataset	No. of genes	No. of samples	Source
Yeast Sporulation	Yeast	474	7	http://cmgm.stanford.edu/pbrown/sporulation
Yeast	Yeast KY	237	18	http://faculty.washington.edu/kayee/cluster/
Yeast	Yeast cell cycle	384	18	http://faculty.washington.edu/kayee/cluster
Human	GDS825	277	8	NCBI
Mouse	GDS958 (Subset)	4000	12	NCBI
Rat	GDS3702 (Subset)	3000	12	NCBI
Rice	Thaliana	517	13	http://homes.esat.kuleuven.be/~sistawww/bioi/thijs/Work/Clustering.html

Characteristics of different real expression datasets used for the experiments along with their sources.

**Figure 6:**
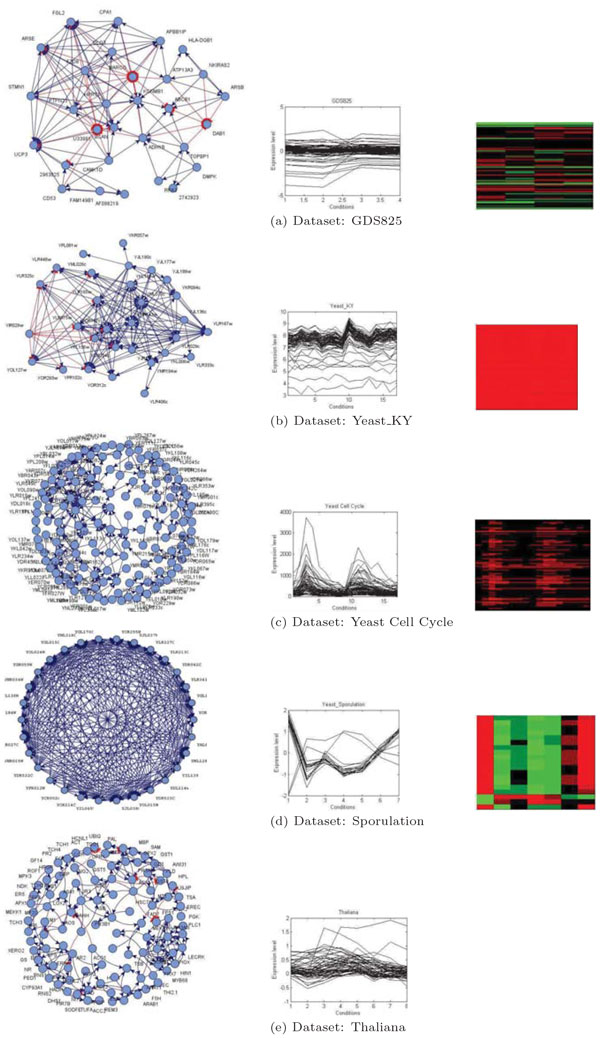
**Network, nodule profile plot and heatmap for each selected module from different datasets Selective network modules from different real datasets are visualized using Marbach platform**. Negative regulations between the genes are represented using red coloured edge and positive regulations are depicted in blue coloured arrow. The profile plots and heatmaps of each module shows the effectiveness of GeCON in detecting both co-regulated and co-expressed network modules.

As discussed, we use the concept of support to draw links or inter-relationships among genes. We hypothesise that two gene expression profiles having more support (positive and negative), i.e. their expression profiles matches more number or cases, more they are biologically related. A gene pair satisfying the support criterion with respect to a user defined threshold *θ *is considered connected. We display only those genes that are linked to others with support higher than the threshold. We use the *in silico *regulatory network construction platform provided by Marbach et. al. [27] for visualizing the networks. In the network, nodes represent genes and lines between nodes represent hypothesized associations among genes. A blue colored arrowhead edge shows positive regulation, whereas a red colored blunt head edge indicates negative regulation between a pair of genes. Some networks we generate are presented in Figure 6. The genes participating in a co-expression network form a group of coherent or co-expressed genes responsible for common biological activities. We consider such a group a module and analyse the biological significance of the modules in terms of Gene Ontology in the next section. Figure 6 also shows the profile plots of selected modules and the corresponding heat map. The largest gene expression values are displayed in red (hot), the smallest values in blue (cool), and intermediate values in shades of red (pink) or blue in the heat map. From the map it can easily be observed that captured modules contain a mix of both up- and down-regulated differentially expressed genes. The cluster profile plot shows the gene expression values of the genes within that cluster with respect to the conditions or time points for each co-expressed group. From the profile, it is evident that GeCON is able to detect both positively and negatively co-regulated gene groups as well as identify scaling and shifting patterns [22] in the expression.

#### Biological significance

We determine the biological relevance of the modules comprising of all the genes participating in a common co-expression network, in terms of *p *[1] and *Q *[31] values against statistically significant GO terms validated using the GO annotation database. For evaluating functional enrichment of a module in terms of *p *values we use FuncAssociate [32]. The *Q*-value is the minimal False Discovery Rate (FDR) at which a gene appears significant. The GO categories and *Q*-values from an FDR corrected hypergeometric test for enrichment are obtained using GeneMANIA [33]. *Q*-values are estimated using the Benjamini Hochberg procedure [31]. We report *p *and *Q*-values of selected modules from several datasets. Along with *Q*-values, GeneMania also provides Co-expression, Physical and Genetic interaction scores for the networks. The co-expression percentage indicates the level of similarity in expressions across conditions. On the other hand, the physical interaction percentage shows the level of protein-protein interaction within a module. In Table 4, we present results from GeneMANIA for selected modules.

**Table 4:** *Q*-value, co-expression and physical interaction score for different modules from different datasets

Dataset	Module No	GO Annotation	Q Value	Co-expression (%)	Physical Interaction (%)
	1	cytosolic ribosome	**1.11E-47**	74.71	7.24
	2	nucleolus	2.32E-30	72.46	8.96
Sporulation	3	sporulation	9.87E-20	**96.96**	0
	4	DNA replication preinitiation complex	2.92E-09	3.07	**95.08**

	1	cytosolic ribosome	**2.16E-130**	69.1	3.56
Yeast KY	2	structural constituent of ribosome	2.64E-126	69.1	3.56
	3	DNA-dependent	2.38E-27	65.05	8.08

		DNA replication			
	1	mitochondrial inner membrane	8.29E-07	68.5	5.41
	2	oxidoreductase activity	3.29E-02	**100**	
GDS3702	3	aging regulation of	1.55E-01	**100**	
	4	lipid catabolic process	1.40E-03	**100**	
	5	iron-sulfur cluster binding	5.51E-03	43.75	9.72

	1	vacuolar proton-transporting	4.67E-16	27.59	32.75
GDS958		V-type ATPase complex			
	2	cell cortex	5.01E-03	27.59	32.75

	1	negative regulation of cellular process	2.19E-04	29.41	29.41
Thaliana	2	response to wounding	1.36E-08	**92.48**	5.63
	3	receptor binding	2.49E-03	29.41	29.41

Statistical significance of selected network modules from real datasets are shown with respect to Q-value based on GO database.

Module 1 obtained from the Yeast Sporulation network is mainly responsible for cytosolic ribosome formation with *Q*-value 1.11e-47 and module 3 exhibits **96.96**% of co-expression where the module is responsible for sporulation in yeast. On the other hand, module 4 is responsible for DNA replication and preinitiation complex formation and shows very high protein-protein interaction of 95.08%. Kayee's Yeast dataset shows a very high *Q*-value of **2.16E-130**. However, the same module shows very poor physical interaction. We also observe 100% co-expression from GDS3702 where module 2 (*Dad1, BI281185, Eif4h, Gnb1, Ahcy, Dpyd, Aldh3a1, Pex6 *), module 3 (*Eif4a3, Psmc2, Cat, Pick1, Zranb2, Erap1, Sacm1l*) and module 4 (*AI411286, Mrps26, Pim3, Thra, Uso1, Apcs, Cacna1a, Pfn2, Ptp4a2, Hrsp12*) are responsible for oxidoreductase activities, aging regulation and lipid catabolic process. The modules extracted from Mouse (GDS958) are responsible for vacuolar proton-transporting V-type ATPase complex formation and cell cortex formation. We also observe **92.48**% of co-expression in the Thaliana network module.

Table 5 presents *p*-value obtained by FuncAssociate for selected modules submitted from different datasets.

**Table 5:** *p*-values for different modules from different datasets

Dataset	Module	GO Annotation	*p *value
	1	folic acid and derivative biosynthetic process	**3.10E-15**
GDS825	2	cullin-RING ubiquitin ligase complex	5.40E-08
	3	chemoattractant activity	5.60E-07
	4	biotin binding	8.30E-07

Yeast KY	1	cytosolic ribosome	**5.20E-96**
	2	DNA replication	9.64E-20

	1	response to neutrient	1.47E-05
GDS3702	2	hydrolase activity	1.60E-05
	3	protein complex	8.00E-04

	1	intracellular part	**9.83E-19**
GDS958	2	intracellular membrane-bounded organelle	2.57E-05

	1	cytoplasmic translation	**2.22E-22**
	2	anatomical structure formation	1.25E-17
Sporulation	3	ribonucleoprotein complex	1.07E-10
	4	cell cycle phase	2.36E-06
	5	cellular component assembly	4.66E-06

Significant modules are shown based on p-score and GO terms.

For Kayee's dataset, GeCON shows better performance in terms of high enrichment with *p*-value, e.g., a *p*-value of **5.20E-96**. Similarly, GDS825, GDS958 and Sporulation datasets also contain modules with good functional enrichments.

## Conclusion

In this paper, we present an effective gene co-expression network finding algorithm called GeCON for discovering biologically related gene pairs that may form a network of co-expressed genes. The GeCON algorithm exploits a fast correlogram matrix based technique for capturing the support for each gene pair in order to compute relationships between gene pairs. Gene pairs with strong relationship are used to construct the network. When constructing networks, GeCON exploits regulation relationships among genes. We report results to show that GeCON is effective in predicting *in slico *networks based on the DREAM Challenge data. We provide results to show that network modules extracted have high biological significance. Moreover, we further establish that the simple expression pattern matching is helpful in finding biologically relevant genes. Gene co-expression networks can be used further to predict more complex biological networks. Work is underway to discover gene regulatory networks with causality information.

## Methods

Global similarity measures such as Euclidean distance or Pearson correlation coefficient may not always capture true gene-gene relationships [34]. In addition, most existing techniques give low emphasis to pattern matching based on local similarity. It has also been observed that genes share local rather than global functional similarity in their gene expression profiles [8]. Moreover, another observation is that most existing techniques are computationally expensive. In this section, we develop an approach based on local expression pattern similarity, to construct co-expression networks with signed edges to represent regulatory relationships among genes. In general, comparing pair-wise gene profiles requires multiple passes over the database, which often is quite expensive, especially for datasets with large numbers of genes. In this work, we perform pair-wise comparison using a one-pass approach, and we compute supports using a single scan of the dataset. Pairs of genes showing similarity above a user-defined threshold *θ *are used to construct the adjacency matrix which is used, in turn, to construct and visualize the network. A preliminary version of the work can be found in [35].

To capture the patterns in an expression profile, we consider the line between two consecutive expression values, termed as *edge*. Thus, for an expression data with *M *conditions or time points, there are (*M *− 1) edges. To represent the edge we use two measures, degree of fluctuation and regulation pattern of the edge. The degree of fluctuation of an edge is the angular deviation of the edge on the 180-degree normal plane. Regulation pattern represents the up- and down-regulation of an edge. The method is discussed in details below.

### Capturing expression patterns

Now, we discuss the preprocessing steps involved in capturing the degree of fluctuation and regulation pattern information for each expression profile. We compare two gene expressions both in terms of degree of fluctuation [36] and pattern of regulation between two adjacent conditions (edges), simultaneously [26]. To capture both regulation pattern and degree of fluctuation of each gene, we read rows of original data with *M *expression values or conditions and convert them into another row of (*M *− 1) columns, each column of which contains the degree of fluctuation and the regulation pattern of an edge between two adjacent conditions. We represent regulation information as 1 and -1 to denote up-regulation and down-regulation, respectively. The regulation value in the *k^th ^*edge of a gene *G_i_, G_i_*(*r*_k_), based on two consecutive conditions (say, *O*_k−1_ &*O*_k_) is calculated as:

(4) $$G_{i}\left(r_{k}\right)=\left\{\begin{array}{c} 1\;\;\text{if}\;O_{k-1}<O_{k}\\ -1\;\;\text{if}\;O_{k-1}>O_{k}. \end{array}\right)$$

To calculate the degree of fluctuation for *k^th ^*edge of *G*_i_, *G*_i_(*a*_k_), we compute the arctangent between two adjacent expression levels (*O*_k−1_, *O*_k_) corresponding to the *k^th ^*edge. We use two argument arctangent function *arctan2*. The purpose of using two arguments instead of one is to gather information on the signs of the inputs in order to return the appropriate quadrant of the computed angle, which is not possible for the single-argument arctangent function. Since, *arctan2 *returns value in the range *−π *to *π*, we convert the angle to be in the 180 degree plane as follows:

(5) $$G_{i}\left(a_{k}\right)=\left\{\begin{array}{c} 180-abs\left(arctan2\left(O_{k},O_{k-1}\right)\right)\;\text{if }O_{k}<O_{k-1}\\ abs\left(arctan2\left(O_{k},O_{k-1}\right)\right)\;\;\;\;\text{otherwise}. \end{array}\right)$$

**Figure 7:**
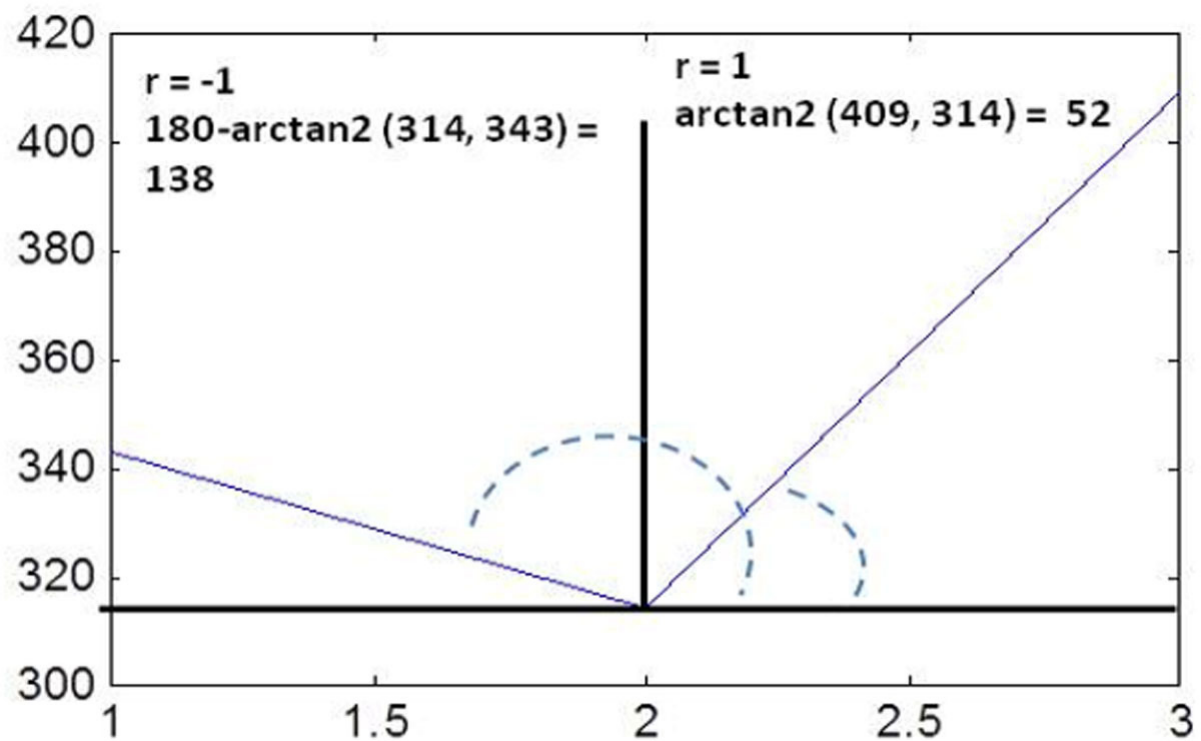
**Degree of fluctuation for three expression values of a gene**. An illustration of converting the expression values of an expression profile in terms of angular deviation and regulation pattern of *edge *between two consecutive expression values.

The fact is illustrated in Figure 7 taking an example of a gene expression dataset with a single gene, *G *= {343, 314, 409} with three expression values. After transforming the values into angular deviation and regulation pattern, it becomes *G *= {138, −1; 52, 1}.

To formulate the pattern similarity based co-expression networking problem we define the following terms based on angular deviation and regulation pattern of a gene expression profile.

### Terminologies used

Let *G *= {*G*_1_, *G*_2_, *· · · , G*_*N*_} be the set of *N *genes and *T *= {*T*_1_, *T*_2_, *· · · , T*_*M*_} be the set of *M *conditions or time points of a microarray dataset. The gene expression dataset *D *is represented as an *N *× *M *matrix *D*_N ×M _where each entry *d*_*i,j*_corresponds to the logarithm of the relative abundance of *mRNA *of a gene. The following definitions and lemmas provide the theoretical basis for the proposed GeCON algorithm.

**Definition 1 **(Pattern Similarity). Given degrees of fluctuation *A *= {*a*_1_, *a*_2_, *· · · , a*_*M *−1_} and regulation patterns *R *= {*r*_1_, *r*_2_, *· · · , r*_*M *−1_} of a gene, derived from the gene expression profile, two gene *G_i _*and *G_j _*s' *k^th ^*expression patterns, *G_ik _*and *G_jk_*, are similar if the difference in the degrees of fluctuation of the two genes' *k^th ^*edges (*G*_*i*_(*a*_*k*_) and *G*_*j*_(*a*_*k*_)) is less than some given threshold *τ*.

In calculating similarity between two genes, we consider two patterns: positive similarity, *Pos_sim*, when the regulation patterns are the same (in case of up-regulation) and negative similarity, *Neg_sim*, when the patterns are inverted (in case of down-regulation) for a particular edge (inverted pattern). Both the similarities are defined as follows:

(6) $$Pos\text{\_}sim\left(G_{ik},G_{jk}\right)=\left\{\begin{array}{c} 1,\;\text{if }G_{i}\left(r_{k}\right)=G_{j}\left(r_{k}\right)\\ \;\;\text{and}|G_{i}\left(a_{k}\right)-G_{j}\left(a_{k}\right)|\;<\tau \\ 0,\;\text{otherwise}, \end{array}\right)$$

(7) $$Neg\text{\_}sim\left(G_{ik},G_{jk}\right)=\left\{\begin{array}{c} 1,\;\text{if }G_{i}\left(r_{k}\right)=-G_{j}\left(r_{k}\right)\\ \;\;\text{and}\\ \;\;|180-G_{i}\left(a_{k}\right)+G_{j}\left(a_{k}\right)|\;<\tau \\ 0,\;\text{otherwise}, \end{array}\right)$$

where *G_i_*(*r_k_*) and *G_j_*(*r_k_*) are the regulation value of *k^th ^*edges of gene *G_i _*and *G_j _*respectively. In case of *Neg_sim*, we subtract 180 from the sum of degree of fluctuation values of *G_i _*and *G_j _*to keep the difference in the range of 0 to 180.

**Definition 2 **(Support). It is the ratio between the number of edges for which genes *G_i _*and *G_j _*are similar and the total number of edges i.e. (*M *− 1). We consider both positive and negative supports to measure the number of edges where both genes have similar or inverted pattern tendencies, respectively. The formulas are given below.

(8) $$Pos\text{\_}support\left(G_{i},G_{j}\right)=\overset{M-1}{\underset{k=1}{\sum }}Pos\text{\_}sim\left(G_{ik},G_{jk}\right)/\;)\left(M-1\right)$$

(9) $$Neg\text{\_}support\left(G_{i},G_{j}\right)=\overset{M-1}{\underset{k=1}{\sum }}Neg\text{\_}sim\left(G_{ik},G_{jk}\right)/)\left(M-1\right)$$

**Definition 3 **(Strongly Connected). Two genes *G_i _*and *G_j _*are said to be *StronglyConnected *(or have an inter-relationship) if *Pos_support*(*G_i_, G_j_*) + *Neg_support*(*G_i_, G_j_*) >*θ*, where *θ *is a user defined threshold to indicate that the minimum number of edges of two expression profiles must match.

**Definition 4 **(Co-expression Network). A Co-expression network is a graph *T *= {*G*', *E*} containing a subset of genes that are strongly connected. If two genes (*G_i_, G_j_*) ∈ *G*' are connected by an arc *E_ij _*∈ *E*, then *G_i _*and *G_j _*are strongly connected to each other. Here, *E *= {(*E_ij_, S_k_*), *· · · *(*E_mn_, S_k_*)} is a set of pairs, where *E_ij _*represents an arc connecting *G_i _*and *G_j_*, and *S_k _*represents the sign of the arc *E_ij_*. A value of *S_k _*= +1 indicates up or positive regulation and -1 indicates down or negative regulation. To calculate the value of *S_k _*of edge *E_ij_*, we use *Pos_support *and *Neg_support*. This is defined as:

(10) $$S_{k}\left(E_{ij}\right)=\left\{\begin{array}{c} +1,\;\text{if }Pos\text{\_}support\left(G_{i},G_{j}\right)>\theta \\ -1,\;\text{if }Neg\text{\_}support\left(G_{i},G_{j}\right)>\theta . \end{array}\right)$$

**Lemma 1**. For any two genes G_i _and G_j_, if G_i _∈ T, a gene co-expression network, and G_i _is strongly connected to G_j_, then G_j _∈ T.

*Proof*. The lemma can be proved by contradiction. Assume, *G_i _*and *G_j _*are two strongly connected genes and *G_j _*∈ *T*, but *G_j _*∉ *T*. As per Definition 4, *T *is a subset of strongly connected genes and since *G_i _*and *G_j _*are strongly connected, *G_j _*∈ *T*, which is a contradiction and hence the proof.   □

Similarly the following lemma is trivial based on the Definitions 1 through 4 and Lemma 1.

**Lemma 2**. Let G_i _and G_j _be two genes, and T_1 _and T_2 _be two gene co-expression networks. If G_j _∈ T_1 _and G_j _∈ T_2_, then G_i _and G_j _are not connected.

**Lemma 3**. Genes belonging to the same gene co-expression network are co-regulated or similar.

*Proof*. This lemma can also be proved by contradiction. Let us assume that any two genes *G_i _*and *G_j _*∈ *T *are not co-expressed. If *G_i _*and *G_j _*are in the same network, they are strongly connected (as per Definitions 3 and 4), and hence *G_i _*and *G_j _*are strongly connected. Again, any two strongly connected genes are similar or co-expressed (as per Definitions 1 through 3), which contradicts the assumption, hence the proof.   □

Similarly, the proof of the following lemma (the reverse case of lemma 3) is trivial.

**Lemma 4**. Genes belonging to different gene networks are not co-expressed.

### Construction of co-expression network

This section discusses the counting of pair-wise support between genes using only *one pass *over the database to construct the co-expression network of connected genes. We use a correlogram matrix approach [37] for computing similarity between two target genes based on the degree of fluctuation and regulation between them. Later, similarity values are used to calculate the support values needed to construct the co-expression network. We first invert the preprocessed database obtained using the above technique, by placing edges as rows and genes as columns. We read each row from the database, and check whether two consecutive genes (say, *G_i _*and *G_j_*) satisfy the similarity criterion (in terms of degree of fluctuation and regulation information) or not, using (6) and (7). If two genes are similar, the content of the correlogram matrix cell with index (*i,j*) is increased. This step is repeated for all pairs of genes for each row. This continues for all the rows to be processed.

From the correlogram matrix, it is very simple to extract the support count of gene pairs. Using these support counts, we compute all strongly connected genes that satisfy the given *θ *constraints. Based on all strongly connected pairs, the adjacency matrix is computed as:

(11) $$A\left(i,j\right)=\left\{\begin{array}{c} +1\;\text{if }G_{i}\text{ and }G_{j}\text{ are strongly connected and }S_{k}\left(E_{ij}\right)=+1\\ -1\;\text{if }G_{i}\text{ and }G_{j}\text{ are strongly connected and }S_{k}\left(E_{ij}\right)=-1\\ \;\;0\;\text{otherwise} \end{array}\right)$$

where 0 indicates the lack of any relation between the genes. A gene co-expression network connecting various genes is constructed using the adjacency matrix.

Our approach is advantageous because (i) it requires only single scan over the database; (ii) it is faster, (iii) our approach does not use any standard proximity measures, (iv) since it is pattern based, it is insensitive to normalization of data as normalize data maintain similar pattern or tendency with original data even after normalization and (v) it does not require any discretization step where continuous values are mapped into pre-specified intervals or classes. The preprocessing steps discussed above are only for an internal representation of expression profile into angular deviation and regulation pattern. Apparently regulation pattern calculation looks like discretization step. However, regulation values, +1 and -1, are simply a symbolic representation of upward and downward inclination of an edge between two consecutive expression values that helps only in choosing appropriate pattern matching formula and calculating *Pos_support *and *Neg_support*. There is no information loss incurred during the conversion.

### GeCON: the algorithm

The steps in GeCON are given in Algorithm 1. Step 1 of the algorithm, is dedicated to the first phase of the approach, i.e., preprocessing dataset *D *to *D*'. Step 2 deals with construction of the correlogram matrix. In step 3, all connected genes are extracted and the adjacency matrix is constructed. Finally, the algorithm returns the adjacency matrix *A*.

**input **: *D *(Expression Dataset), *θ *(Support threshold)

**output**: A (Adjacency matrix)

**1 **Preprocess original database D to D' wrt. *τ*;

**2 **Generate correlogram matrix from D';

**3 foreach ***gene pair *(*G_i_, G_j_*) ∈ D' **do**

**4 **      Compute all connected gene pairs by using support count from the correlogram matrix wrt. *θ*;

**5 **      Construct adjacency matrix A using all connected genes with regulation information;

6 end

**7 **Return A;

**Algorithm 1: **The GeCON Algorithm

### Complexity analysis

GeCON uses a correlogram matrix for storing support for pairs of genes. Thus for *N *genes, GeCON requires fixed memory of size *N *× (*N *− 1)*/*2. GeCON needs time for preprocessing and network construction using the correlogram matrix. For a dataset with *N *genes and *C *conditions, the preprocessing step requires *O*(*N *∗ *C*) time and to transpose the preprocessed data it requires *O*(*C *∗ *N *) time. To construct the network, it traverses the correlogram matrix. Thus, the time required for network construction is *O*(*N *× (*N *− 1)*/*2). The total computational cost of GeCON is:

$$\begin{array}{cc} Cost_{GeCON} & =O\left(N*C\right)+O\left(C*N\right)+O\left(N\times \left(N-1\right)/2\right)\\ & \approx O\left(N\right)+O\left(N\times \left(N-1\right)/2\right),\left(\text{generally},C\ll N\text{ and so we can ignore }C\right)\\ & \approx O\left(N\times \left(N-1\right)/2\right),\left(\text{which is even}<\;N^{2}/2\right). \end{array}$$

## Availability of supporting data

A Java implementation of GeCON (as executable) and few sample expression datasets used in this paper are available at https://sites.google.com/site/swarupnehu/publications/resources.

## Competing interests

The authors declare that they have no competing interests.

## Authors' contributions

SR: Conceived the idea, conducted research, designed study, participated in design of algorithm, wrote manuscript. DKB: Participated in design of algorithm, supervised research, wrote manuscript. JKK: Supervised research, wrote manuscript. All authors read and approved the final manuscript.

## Acknowledgements

The authors would like to thank the anonymous reviewers and the editor of this journal for their constructive suggestions.

## Declarations

Publication of this article was funded by the authors and in part by DeitY, Govt. of India.

This article has been published as part of *BMC Bioinformatics *Volume 15 Supplement 7, 2014: Selected articles from the 10th Annual Biotechnology and Bioinformatics Symposium (BIOT 2013). The full contents of the supplement are available online at http://www.biomedcentral.com/bmcbioinformatics/supplements/15/S7
